# Blood-Feeding Induces Reversible Functional Changes in Flight Muscle Mitochondria of *Aedes aegypti* Mosquito

**DOI:** 10.1371/journal.pone.0007854

**Published:** 2009-11-16

**Authors:** Renata L. S. Gonçalves, Ana Carolina L. Machado, Gabriela O. Paiva-Silva, Marcos H. F. Sorgine, Marisa M. Momoli, Jose Henrique M. Oliveira, Marcos A. Vannier-Santos, Antonio Galina, Pedro L. Oliveira, Marcus F. Oliveira

**Affiliations:** 1 Laboratório de Bioquímica Redox, Instituto de Bioquímica Médica, Programa de Biologia Molecular e Biotecnologia, Universidade Federal do Rio de Janeiro, Rio de Janeiro, Rio de Janeiro, Brazil; 2 Laboratório de Inflamação e Metabolismo, Instituto Nacional de Ciência e Tecnologia de Biologia Estrutural e Bioimagem (INBEB), Universidade Federal do Rio de Janeiro, Rio de Janeiro, Rio de Janeiro, Brazil; 3 Laboratório de Bioquímica de Artrópodes Hematófagos, Instituto de Bioquímica Médica, Programa de Biologia Molecular e Biotecnologia, Universidade Federal do Rio de Janeiro, Rio de Janeiro, Rio de Janeiro, Brazil; 4 Instituto Nacional de Ciência e Tecnologia em Entomologia Molecular (INCT-EM), Rio de Janeiro, Brazil; 5 Laboratório de Biologia Molecular de Plantas, Instituto de Bioquímica Médica, Programa de Biologia Molecular e Biotecnologia, Universidade Federal do Rio de Janeiro, Rio de Janeiro, Rio de Janeiro, Brazil; 6 Laboratório de Biomorfologia Parasitária, Instituto de Pesquisa Gonçalo Moniz, Fiocruz, Salvador, Bahia, Brazil; 7 Laboratório de Bioenergética & Fisiologia Mitocondrial, Instituto de Bioquímica Médica, Programa de Bioquímica e Biofísica Celular, Universidade Federal do Rio de Janeiro, Rio de Janeiro, Rio de Janeiro, Brazil; Universidade de Brasília, Brazil

## Abstract

**Background:**

Hematophagy poses a challenge to blood-feeding organisms since products of blood digestion can exert cellular deleterious effects. Mitochondria perform multiple roles in cell biology acting as the site of aerobic energy-transducing pathways, and also an important source of reactive oxygen species (ROS), modulating redox metabolism. Therefore, regulation of mitochondrial function should be relevant for hematophagous arthropods. Here, we investigated the effects of blood-feeding on flight muscle (FM) mitochondria from the mosquito *Aedes aegypti*, a vector of dengue and yellow fever.

**Methodology/Principal Findings:**

Blood-feeding caused a reversible reduction in mitochondrial oxygen consumption, an event that was parallel to blood digestion. These changes were most intense at 24 h after blood meal (ABM), the peak of blood digestion, when oxygen consumption was inhibited by 68%. Cytochromes *c* and *a*+*a*_3_ levels and cytochrome *c* oxidase activity of the electron transport chain were all reduced at 24 h ABM. Ultrastructural and molecular analyses of FM revealed that mitochondria fuse upon blood meal, a condition related to reduced ROS generation. Consistently, BF induced a reversible decrease in mitochondrial H_2_O_2_ formation during blood digestion, reaching their lowest values at 24 h ABM where a reduction of 51% was observed.

**Conclusion:**

Blood-feeding triggers functional and structural changes in hematophagous insect mitochondria, which may represent an important adaptation to blood feeding.

## Introduction

Mitochondria are organelles involved not only on aerobic energy transduction from nutrient oxidation to allow ATP synthesis through the oxidative phosphorylation, but also in redox balance, representing one of the major sources of cellular reactive oxygen species (ROS). A small portion of the oxygen consumed by mitochondria is partially reduced to superoxide (O_2_• ^−) radicals and to hydrogen peroxide (H_2_O_2_) [1], [2], which diffuses through the cell playing both signaling and harmful roles [2]. Essentially, O_2_• ^−radicals are generated within the mitochondrial matrix, at the intermembrane space and the outer membrane, which are then dismutated to H_2_O_2_ by superoxide dismutases [2]. In insects, an additional mechanism involved in mitochondrial O_2_• ^− production is the activity of glycerol 3-phosphate dehydrogenase (G3PDH) [3], [4]. Mitochondrial ROS generation is highly regulated and depends on different factors such as the i) electron flux through the inner membrane, ii) the magnitude of the mitochondrial membrane potential (ΔΨ_m_), iii) the oxygen tension, iv) substrate availability, v) NADH/NAD^+^ ratio in the matrix and vi) mitochondrial morphology [1]–[8]. Mitochondria are very dynamic organelles that may fuse or divide, changing their morphology in response to many different stimuli [9]. In this regard, mitochondrial fission is being correlated to increased ROS generation whereas fusion has been associated to protective events [10] leading to a decrease in H_2_O_2_ production [6], [7].

Flying insects have been used as models to study energy metabolism because the FM has the highest respiratory activity among all animal tissues and possess giant mitochondria [11]. In insects, oxygen is delivered to cells through a highly branched system, called tracheae, which open externally in valve-like structures responsible to control gas exchange. The tracheolar invaginations are branched in such a way that their finest branches lie adjacent to tissues, exposing them to a much higher oxygen concentration when compared to oxygen levels in mammalian tissues [12], [13]. The opening of the tracheolar system is tightly regulated by the oxygen availability and it seems to act as a preventive antioxidant mechanism regulating gas exchange during resting/activity cycles [13]. Another point that attracts much interest is that mitochondrial ROS generation seems to play a key role in respiratory capacity and aging [14]–[16]. There is strong evidence linking the chronic accumulation of oxidatively damaged biomolecules to a decrease in respiratory capacity and energy transduction in mitochondria during the aging process [14], [17]. Mitochondria-generated reactive species are central players in this process [18] and long-term reduction in quantity and quality of the ingested food is the only intervention known to increase life span from worms and yeasts to mammals [19]–[22].

Blood-feeding insects are vectors of several important infectious diseases, such as leishmaniasis, malaria, dengue and yellow fever. Additionally, hematophagous insects rely, at least in a part of their life-cycle, on the ingestion of a considerable amount of vertebrate blood to meet their energy demands as well as to drive oogenesis [23], [24]. The threat of oxygen toxicity is especially critical to blood-feeding insects, as, in addition to the oxidative challenge resulting from the ROS produced by mitochondrial metabolism, they have to cope with a powerful pro-oxidant diet composed mainly of hemoglobin and its highly reactive digestion products, heme and iron [25].

Here, we investigated the effects of blood feeding on functional and structural mitochondrial parameters obtained from the FM of *Aedes aegypti* female mosquitoes. We observed that blood feeding caused a transient reduction in oxygen consumption that is correlated with reduced cytochromes *c* and *a* + *a_3_* levels and reduced cytochrome *c* oxidase activity. Interestingly, blood feeding also promoted mitochondrial fusion, a condition related to reduced ROS generation. Consistently, we observed that blood feeding induced a reversible decrease in mitochondrial H_2_O_2_ formation during blood digestion. We speculate that mitochondrial remodeling triggered by blood feeding may represent an important adaptive mechanism for hematophagous insects.

## Materials and Methods

### Ethics Statement

All animal care and experimental protocols were conducted following the guidelines of the institutional care and use committee (Committee for Evaluation of Animal Use for Research from the Federal University of Rio de Janeiro, CAUAP-UFRJ) and the NIH Guide for the Care and Use of Laboratory Animals (ISBN 0-309-05377-3). The protocols were approved by CAUAP-UFRJ under the registry #IBQM001. Technicians dedicated to the animal facility at the Institute of Medical Biochemistry (UFRJ) carried out all aspects related to rabbit husbandry under strict guidelines to insure careful and consistent handling of the animals.

### Insects

*Aedes aegypti* (Red eyes strain) were maintained at 28°C, 70–80% relative humidity with a photoperiod of 12 h light/dark (L:D, 12∶12 h). Larvae were reared on a diet consisting of commercial dog chow. Adults were kept at the same temperature, humidity and photoperiod. On average 200 mosquitoes were placed in 5 L cages. After moulting, adult female mosquitoes were allowed to feed *ad libitum* on pads soaked with 10% (w/v) sucrose solution. Two experimental groups were designed to determine whether *A. aegypti* mitochondrial function would be affected by blood-feeding. In one group, the sugar-fed (SF) female adult mosquitoes 2–5 days after emergence were allowed to feed *ad libitum* only on the pads soaked with sucrose. The other group were blood-fed (BF) female adult mosquitoes 2–5 days after emergence that, besides sucrose, were also allowed to feed on rabbit blood. The mosquitoes were allowed to feed for 30 minutes directly on an ear of an immobilized and anesthetized rabbit at the Institute of Medical Biochemistry animal care facility in Federal University of Rio de Janeiro (UFRJ). Then, flight muscle (FM) mitochondrial preparations were obtained from SF and BF insects at different times.

### Mitochondria

Isolation of mitochondria from *A. aegypti* FM was carried out by using a method described in the literature with modifications [3]. About 150 female mosquitoes were immobilized by chilling on ice and then dissected to obtain the thoraxes, which were gently homogenized in a 15 mL Potter-Elvehjem tissue grinder in a Teflon pestle with 10 mL of ice-cold isolation medium (250 mM sucrose, 5 mM Tris-HCl, 2 mM EGTA, 1% (w/v) fatty acid free bovine serum albumin, pH 7.4). The preparation was maintained at 4°C throughout the subsequent washing and centrifugation procedures. The liquid was passed through cotton gauze in order to get rid of mosquito's legs, wings and remains of thoraxes and was immediately centrifuged twice at 300 ×*g* for 5 min in a Eppendorf centrifuge model 5810-R with a rotor F34-6-38. The supernatant was centrifuged at 10,000 ×*g* for 10 min. The brown pellet was carefully re-suspended in approximately 100 µL of respiration medium (120 mM KCl, 5 mM KH_2_PO_4_, 3 mM Hepes, 1 mM EGTA, 1 mM MgCl_2_, and 0.1% fatty acid free bovine serum albumin, pH 7.2), to give preparations with an average protein concentration of 25 mg/mL. Protein concentration was determined by the Lowry method, using bovine serum albumin as standard [26]. Usually this method yields about 8.62 mg mitochondrial protein/g of mosquito thorax.

### Mitochondrial Respiration

The rates of mitochondrial respiration were assessed at different metabolic states [27] by measuring the oxygen consumption in the presence of oxidizable substrates alone (state 2), and supplemented with sequential additions of ADP (state 3), oligomycin (state 4-like) and FCCP (carbonyl cyanide p-trifluoromethoxyphenylhydrazone) (uncoupled) in an oxygraph (Yellow Springs Instruments, model 5300) fitted with Clark-type electrode in a water-jacketed glass chamber or in a high-resolution oxygraph (O2k, Oroboros Inc., Austria). For all the experiments, the temperature was maintained at 27.5°C and the total reaction volume was 1.0 mL for the measurements in the YSI oxygraph and 2.0 mL in the Oroboros oxygraph. Freshly isolated mitochondria (0.44 mg of protein) were added to the respiration buffer and allowed to equilibrate for 30 s. NAD^+^-linked substrates (10 mM pyruvate plus 10 mM proline-pyr/pro) were added to the chamber and allowed to equilibrate, followed by the addition of ADP (1 mM final concentration). The concentration of substrates used to induce ADP-stimulated state 3 respiration were established based on previous experiments showing that, in this condition, oxygen consumption was at its maximum (data not shown). State 4-like respiration was achieved by adding 4 µg/mL oligomycin, and the respiratory control ratio (RCR) was calculated by dividing the state-3 respiratory rates to oligomycin-induced state 4-like respiratory rates. Afterwards, the proton ionophore FCCP (up to 7 µM) was added to induce oxygen consumption in a complete uncoupled state.

### Cytochrome *c* Oxidase (COX) Activity

Activity of cytochrome *c* oxidase (COX) was measured in triplicate at room temperature, in a total reaction volume of 1 mL, using a GBC spectrophotometer UV/VIS 920 (GBC Scientific Equipment, Australia). Enzyme activity was measured by following the decrease in absorbance due to the oxidation of ferrocytochrome *c*, (ε = 18.5 mM^−1^ cm^−1^) [28]. The reaction mixture consisted of 100 mM potassium phosphate, pH 7.4, 50 µM reduced cytochrome *c*. The reaction was initiated by the addition of freeze-thawed mitochondria (70 µg of protein) and the reduction in absorbance at 550 nm was monitored. KCN (1 mM) was added to inhibit cytochrome *c* oxidase activity, which was considered as the cyanide-sensitive rate of cytochrome *c* oxidation.

### Cytochrome Content

*A. aegypti* mitochondrial preparations were diluted to 100 µg/mL in potassium phosphate buffer 100 mM (pH 7.2) and 5% Triton X-100. Light absorption spectra were obtained between 500 nm and 600 nm in a GBC spectrophotometer UV/VIS 920 (GBC Scientific. Equipment, Australia). The oxidized cytochrome spectra were obtained in the presence of 5 mM succinate while for reduced cytochromes spectra, small quantities of sodium dithionite crystals were added to the 1 mL sample cuvettes. The relative amount of cytochrome was calculated by subtracting oxidized spectra from the equivalent reduced ones, using the millimolar absorbance coefficients of 18.5 mM^−1^ · cm^−1^ (for cytochrome *c*) and 16 mM^−1^ · cm^−1^ (for cytochromes *a* + *a_3_*) at 550 nm [28].

### Transmission Electron Microscopy (TEM) and Morphometry

Thoraxes from SF and BF mosquitoes were fixed in electron microscopy-grade 1% glutaraldehyde (Sigma, USA), 4% formaldehyde and 5 mM CaCl_2_ in 0.1 M sodium cacodylate buffer (pH 7.2), postfixed in 1% osmium tetroxide and 0.08% potassium ferricyanide, dehydrated in an acetone series and embedded in Polybed resin (Polysciences Inc., USA). Thin sections were stained with uranyl acetate and lead citrate, and then observed under a Zeiss EM 109 transmission electron microscope. Morphometric analyses of *A. aegypti* FM mitochondria were carried out by TEM using the software AnaliSYS (Soft Imaging System, Germany). To determine mitochondrial density and area, twenty-two fields from BF and twenty-two fields from SF samples, which excluded nuclear areas, were randomly selected. The number of mitochondria per field (n = 58 for SF and n = 41 for BF), as well as their area, were calculated using the magnifications of 7,000 x and 12,000 x. Mitochondrial density was expressed as the number of organelles per 10 µm^2^, whereas mitochondrial area was calculated by measuring absolute mitochondrial size and dividing by the absolute area of myofibrils.

### RNA Extraction and qRT-PCR Analysis

Total RNAs from thoraxes were extracted from SF and BF (24 h) female mosquitoes using the TRIzol® reagent (Invitrogen, USA), following the manufacturer's instructions. RNA was treated with DNAse I (Invitrogen, USA) and first-strand cDNA synthesis was carried out using High-Capacity cDNA Reverse transcritption kit (Applied Biosystems, USA). Quantitative PCR was performed in a 7500 Real Time PCR System (Applied Biosystems, USA) using SYBR-GREEN PCR master MIX (Applied Biosystems, USA). The comparative Ct method was used to evaluate changes in gene expression levels [29]. The *A. aegypti* ribosomal protein 49 gene *RP-49* was used as endogenous control (accession number AAT45939), according to previous methods [30]. Analysis of expression of the two orthologous genes related to mitochondrial fusion mitofusin (*Mfn*, accession number XM_001649176) and *optic atrophy 1* (*OPA1*, accession number XM_001657256) was carried out by using the following primers: *Mfn:* MFN-F 5′ CCACACGGAAGCTGAAGTTG 3′and MFN-R 5′-ATCGACAACGCGACACAATC 3′; *OPA1:* OPA-F 5′-CTGGCGTATCCAACAGGTGA 3′ and OPA-R 5′-TTCTCGTCGTCTTCGCCATA 3′ ; *RP49:* RP49-F 5′-GCTATGACAAGCTTGCCCCCA 3′ and RP49-R 5′-TCATCAGCACCTCCAGCT 3′. The relative quantification of *Mfn* and *OPA1* was determined using the comparative Ct method, also known as the ΔΔCt method, or the 2T-ΔΔCt method [29], having the constitutive gene *RP49* as the endogenous control. Standard curves were constructed for *Mfn*, *OPA1* and *RP49* primers to validate the application of the comparative Ct method. Data analysis was carried out using Excel software (Microsoft, USA) and statistics were performed using ΔCt values.

### Hydrogen Peroxide (H_2_O_2_) Release

H_2_O_2_ production was assessed by monitoring resorufin fluorescence due to the oxidation of 5 µM amplex red (Invitrogen, USA) in the presence of 1.0 unit/mL horseradish peroxidase (Sigma, USA). The incubation media contained the “respiration buffer” and 10 mM pyruvate and 10 mM proline. The rate of amplex red oxidation was recorded at room temperature using a Cary Eclipse spectrofluorimeter (Varian, USA) adapted with a continuous stirring device, operating at excitation and emission wavelengths of 530 nm and 590 nm, respectively. After each measurement, a standard curve of reagent grade H_2_O_2_ (Merck, Germany) was performed. The “absolute” levels of H_2_O_2_ detected flurorimetrically through this and other methodologies reflect the balance between the generated and detoxified H_2_O_2_ since mitochondria has their own antioxidant defences.

### Data and Statistics

Data in graphs were presented as bars with mean ± SEM values for each condition. Comparisons between groups were done by one-way ANOVA and *a posteriori* Tukey's test for pair-wise comparisons. When appropriate, unpaired Student's t-tests were employed. Differences of *p*<0.05 were considered to be significant. Student's t-test, ANOVA and Tukey's *a posteriori* tests were performed using GraphPad Prism version 4.00 for Windows (GraphPad Software, USA).

## Results

### *A. aegypti* FM Mitochondria Use both NAD^+^ and FAD^+^-Linked Substrates

In agreement with data obtained from other insects [3], [14], [31], *A. aegypti* FM mitochondria from SF or BF mosquitoes were able to oxidize the NAD^+^-linked substrates, pyruvate and proline (pyr/pro), and also the FAD^+^-linked substrate, glycerol 3-phosphate (G3P) (Figure S1). In SF mosquitoes, there were no differences in the mean oxygen consumption rates between pyr/pro and G3P in both ADP-induced (state 3) (*p* = 0.295) and FCCP-induced (uncoupled) (*p* = 0.105) respiration (compare Figure S1A and S1B full lines and Table 1). This result is in agreement with the recently reported findings of Giulivi and colleagues and re-inforces the idea that proline and G3P-induced oxygen consumption occurs in a similar extent in insects [31]. Oligomycin-induced state 4-like respiration in SF mosquitoes using G3P was significantly higher (*p*<0.0001) when compared to pyr/pro (Figure S1A and S1B – dashed lines and Table 1). Also, the differences between state 3 and state 4-like respiratory rates were more evident when NAD^+^-linked substrates were used, as previously reported for *Drosophila* [3], [14]. Consequently, the ratios between state 3 and state 4-like respiratory rates (the respiratory control ratio, RCR), a parameter that measure the integrity of inner mitochondrial membrane, were significantly higher for pyr/pro compared to G3P (9.53±1.06 *vs.* 1.87±0.37, *p*<0.0001, see Table 1).

**Table 1:** Effects of blood-feeding on oxygen consumption rates in different metabolic states in *A. aegypti* FM mitochondria.

	pyruvate +proline	ADP	Oligo	FCCP	RCR
**SF**	17.81±2.02 (n = 13)	139.01±15.52 (n = 13)	14.58±1.18 (n = 13)	114.5±9. 57 (n = 8)	9.53±1.06 (n = 13)
**BF**	7.64±1.24 (n = 14) *b*	43.47±5.09 (n = 14) *b*	6.57±1.25 (n = 13) *b*	44.21±8.55 (n = 14) *b*	6.6±0.78 (n = 14) *a*
% reduction (to SF)	57,10%	68,70%	54,90%	61,40%	30,70%

	Glycerol-3 phosphate	ADP	Oligo	FCCP	RCR
**SF**	104.2±12.48 (n = 7)	175.9±35.78 (n = 7)	93.97±13.8 (n = 7)	185.5±45.58 (n = 6)	1.87±0.38 (n = 7)
**BF**	70.4±9.3 (n = 8) *a*	101.15±25.17 (n = 8)	56.44±3.8 (n = 8) *a*	125.68±7.32 (n = 8)	1.73±0.37 (n = 8)
% reduction (to SF)	32,80%	42,50%	39,90%	32,30%	7,41%

Values were expressed as mean ± SEM of nmols oxygen consumed/min/mg protein in four different mitochondrial metabolic states using: pyruvate + proline or Glycerol-3 phosphate, followed by the addition of 1 mM ADP (ADP), 4 µg/mL oligomycin (Oligo) and 5–7 µM FCCP. RCR (respiratory control ratios) were calculated by dividing the rates of oxygen consumption in state 3 (ADP-stimulated) by those of state 4 (in the presence of oligomycin). The inhibition of oxygen consumption promoted by blood feeding were expressed as the percentage relative to their respective metabolic states in SF. SF  =  sugar fed; BF  =  24 h ABM. Statistical analyses between groups were performed by using the Student's t-test. *a*, *p*<0.05; *b*, *p*<0.0001 both relative to SF.

### Mitochondrial Oxygen Consumption Is Reduced upon Blood Feeding

The effects of blood feeding on *A. aegypti* FM mitochondrial respiration were first assessed at 24 h ABM, a time point considered by the literature to be the peak of blood digestion [32]. At this time, we observed a reduction in oxygen consumption in all mitochondrial metabolic states respiring both pyr/pro and G3P (Figure S1A and S1B, compare full with dashed lines and Table 1). These changes were more pronounced when mitochondria oxidize pyr/pro (Table 1), where significant differences between SF and BF mosquitoes were detected in all four metabolic states analyzed (*p*<0.0001). In fact, the reduction observed at 24 h ABM with pyr/pro-induced respiration in both state 3 and uncoupled (FCCP) (68.7% and 61.4% respectively) respiratory rates were much higher than with G3P (42.5% and 32.2%, respectively). Therefore, a significant change in the respiratory control ratio (RCR) was only observed when pyr/pro were used as substrates, mainly due to a more potent decline in the state 3 respiratory rates. We also observed that in BF insects addition of the uncoupler FCCP did not restore the electron flow and oxygen consumption rates to levels compared to those exhibited by SF insects, suggesting that the phosphorylation apparatus (F_1_F_o_-ATP synthase, Adenine Nucleotide Translocator and the phosphate carrier) were not involved in this phenomena. In fact, blood feeding did not affect the mitochondrial azide-sensitive ATPase activity (Figure S2A).

### Blood-Feeding Induces a Time-Dependent Reversible Inhibition of Mitochondrial Oxygen Consumption

It is well known that BF causes important physiological and metabolic changes in *A. aegypti* females [33]–[35]. After a blood meal, proline levels in *A. aegypti* hemolymph reach about 100 mM, being the most abundant aminoacid in this fluid [33] and its oxidation is capable of sustaining flight not only in *A. aegypti* [36] but also in other insects [37]. The respiratory activity sustained by pyr/pro is a direct consequence of the uptake by mitochondria, the activity of pyruvate dehydrogenase, proline oxidase, the enzymes of TCA cycle and the ETC complexes. Since mitochondrial proline oxidation in insects renders higher RCR indexes compared to G3P [3], [14] (Table 1), we further investigated the dynamics of mitochondrial functional changes triggered by blood feeding on *A. aegypti* FM using pyr/pro as substrates. Representative traces of mitochondrial oxygen consumption in SF and BF mosquitoes after 0.25 h, 2 h, 24 h, 40 h and 72 h ABM are shown in Figure S3. Comparing the traces of SF and BF, at different times after blood feeding, we can observe a transient reduction in mitochondrial oxygen consumption rates in all metabolic states, particularly at state 3 and uncoupled respiration (Table 2). Curiously, just 0.25 h after blood intake, a marked decrease in the respiratory rates were observed in all metabolic states, being of 28.1% at state 3 and 26.1% at the uncoupled respiration (Table 2). However, further inhibition of respiration was potentiated up to 24 h ABM, when the highest inhibitory effects of BF on oxygen consumption were detected (68.7% at state 3 and 61.4% at uncoupled respiration) (Table 2). After complete blood digestion, at 72 h ABM, oxygen consumption rates returned to levels found before BF (Figure S3 and Table 2). We conclude that the inhibitory effects of blood feeding on *A. aegypti* FM mitochondrial respiration were parallel to blood digestion. Also, since FCCP was unable to restore the oxygen consumption rates in BF mosquitoes to the levels found in SF insects, a putative underlying mechanism could be the impairment of electron transport through the mitochondrial complexes at the inner membrane.

**Table 2:** Oxygen consumption rates in FM mitochondria from SF and BF *A. aegypti* along the blood digestion process oxidizing pyruvate and proline.

	pyruvate +proline	% red.	ADP	% red.	Oligo	% red.	FCCP	% red.	RCR	% red.
**SF**	17.81±2.02 (n = 13)	_	139.01±15.52 (n = 13)	_	14.58±1.18 (n = 13)	_	114.5±9. 57 (n = 8)	_	9.53±1.06 (n = 13)	_
**BF 0.25 h**	10.85±0.92 (n = 14) *b*	39,10%	99.94±7.08 (n = 17)	28,10%	10.42±1.07 (n = 17)	37,70%	84.57±13.58 (n = 17)	26,10%	10.99±0.78 (n = 17)	0%
**BF 2 h**	9.29±1.04 (n = 16) *c*	47,80%	100.81±13.12 (n = 16)	27,50%	11.45±1.38 (n = 16)	21,50%	59.29±12.90 (n = 15) *a*	48,20%	8.81±1.4 (n = 16)	7,55%
**BF 24 h**	7.64±1.24 (n = 14) *c*	57,10%	43.47±5.09 (n = 14) *c*	68,70%	6.57±1.25 (n = 13) *b*	54,90%	44.21±8.55 (n = 14) *b*	61,40%	6.6±0.78 (n = 14)	30,70%
**BF 40 h**	7.38±0.62 (n = 14) *c*	58,60%	52.85±6.56 (n = 16) *c*	62%	7.97±1.21 (n = 18) *a*	45,30%	n.d.	n.d.	6.63±0.82 (n = 16)	30,40%
**BF 72 h**	15.99±1.41 (n = 15)	10,20%	110.91±6.67 (n = 15)	20,20%	18.96±1.81 (n = 14)	0%	101.62±7.96 (n = 15)	11,20%	5.85±0.35 (n = 15)	38,60%

Oxygen consumption was determined in SF and in BF insects five different times ABM (0.25 h, 2 h, 24 h, 40 h and 72 h). Values were expressed as mean ± SEM of nmols oxygen consumed/min/mg protein in four different mitochondrial metabolic states using: 10 mM pyruvate + proline, followed by the addition of 1 mM ADP (ADP), 4 µg/mL oligomycin (Oligo) and 5–7 µM FCCP. RCR (respiratory control ratios) were calculated by dividing the rates of oxygen consumption in state 3 (ADP-stimulated) by those of state 4 (in the presence of oligomycin). Inhibition of oxygen consumption promoted by BF was expressed as the percentage relative to their respective metabolic states in SF (% red). Statistical analyses between groups were performed by using ANOVA and *a posteriori* Tukey's test. *a*, *p*<0.05; *b*, *p*<0.01; *c*, *p*<0.001 all relative to their respective metabolic states in SF.

### Cytochrome *c* Oxidase Activity and ETC Cytochrome Levels Were Reduced after Blood Feeding

**Figure 1:**
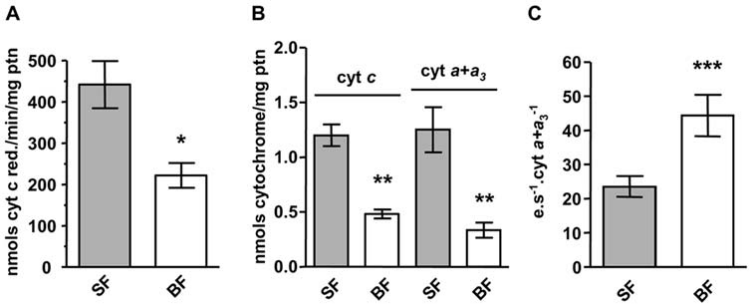
Blood feeding reduces cytochrome *c* oxidase activity and cytochromes content in *A. aegypti* FM mitochondria. Comparison of cytochrome *c* oxidase (COX) activity, cytochrome content and electron turnover number of COX were assessed in sugar-fed (SF, grey bars) and 24 h ABM (BF, white bars) *A. aegypti* FM mitochondria. (A) COX activity was measured as the KCN-sensitive rate of ferrocytochrome *c* oxidation at 550 nm in freeze-thawed mitochondria preparations (SF, n = 21 *vs.* BF, n = 10; * *p* = 0.0157). (B) Quantification of cytochromes *c* (cyt *c*) and *a*+*a*_3_ (cyt *a*+*a*_3_) in fresh mitochondrial preparations (cyt *c*: SF, n = 2 *vs.* BF, n = 3; ** *p*<0.01; cyt *a*+*a*_3_: SF, n = 2 *vs.* BF, n = 3; ** *p*<0.01). (C) Electron turnover number of cytochrome *c* oxidase was obtained in freeze-thawed mitochondria preparations (SF, n = 21 *vs.* BF, n = 10; *** *p* = 0.0017). Bars represent mean ± SEM. Statistical analysis was performed using Student's t-test.

Measurement of mitochondrial ETC complex activity indicates that despite blood feeding did not affect the rotenone-sensitive NADH-induced cytochrome *c* reduction or the antimycin a-sensitive G3P-induced cytochrome *c* reduction (Figure S2B and S2C, respectively) it specifically reduces cytochrome *c* oxidase (COX) activity at 24 h ABM (Figure 1A, *p* = 0.0157). Similar results were obtained when COX activity was assessed by using a mixture of TMPD-ascorbate as substrates (data not shown). Consistently, significant decreases in both cytochrome *c* (*p* = 0.0127) and cytochromes *a* + *a*_3_ (*p* = 0.0139) levels were also observed in BF insects (Figure 1B). Turnover numbers for COX were significantly higher (*p* = 0.0017) in mitochondria isolated from BF mosquitoes suggesting that enzyme inhibition results in a compensatory mechanism in response to a diminished electron flow (Figure 1C).

### *A. aegypti* Mitochondria Fuses upon Blood Meal

**Figure 2:**
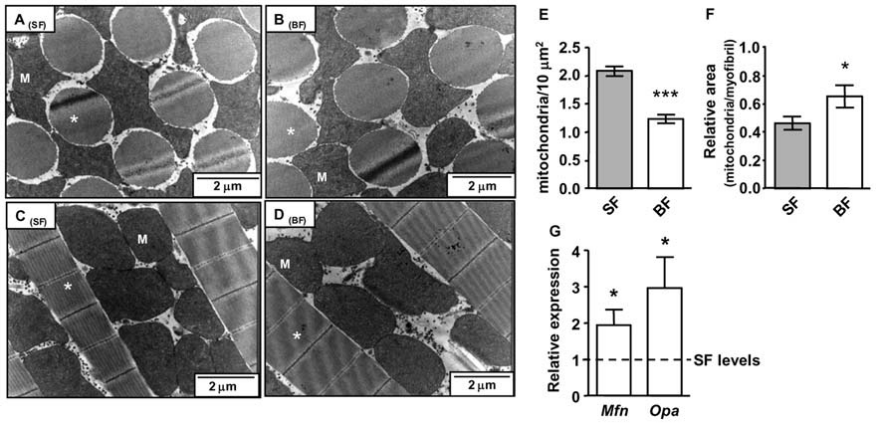
Mitochondrial fusion triggered by blood meal in *A. aegypti* FM. Transmission electron microscopy of transversal sections of *A. aegypti* FM fed on (A) sugar or (B) blood 24 h ABM showing the general architecture of myofibrils (asterisks) and the giant mitochondria (M). Longitudinal sections of *A. aegypti* FM fed on (C) sugar or (D) blood showing the myofibrils (asterisks) and the giant mitochondria (M). Scale bars represent 2 µm. (E) Mitochondrial density in sugar-fed (SF, grey bars) and 24 h ABM (BF, white bars) was calculated by dividing the number of organelles (SF, n = 23 *vs.* BF, n = 18; *** *p*<0.0001) per 10 µm^2^. (F) Relative mitochondrial area in sugar-fed (SF, grey bars) and 24 h ABM (BF, white bars) was calculated by measuring absolute mitochondrial size and dividing by the absolute area of myofibrils (SF, n = 61 *vs.* BF, n = 41; * *p*<0.05). (G) Expression of mitofusin (*Mfn*) and optic atrophy 1 (*OPA1*) mRNA levels evaluated by qPCR in sugar-fed (dashed line) and 24 h ABM (white bars)(* *p*<0.05). Bars represent mean ± SEM and statistical analysis between groups was performed using Student's t-test.

Figure 2 shows transmission electron microscopy (TEM) images of *A. aegypti* FM from transversal (Figures 2A and 2B) and longitudinal (Figures 2C and 2D) sections of SF (Figures 2A and 2C) and BF (Figures 2B and 2D) mosquitoes. The expected organization pattern of muscle fibers, being wrapped with large mitochondria presenting highly packed cristae, is clearly seen in all images as has been described for other insects [11]. The gross organization and structure of *A. aegypti* FM mitochondria did not change at 24 h ABM, seen both in transversal (compare Figures 2A and 2B) and longitudinal sections (compare Figures 2C and 2D). However, significant quantitative morphological changes were observed after a blood meal, such as a 41% decrease in mitochondrial density (number of mitochondria per field area, Figure 2E, *p*<0.0001). Parallel to this, we also observed an increase of about 46% in mitochondrial size, expressed as mitochondrial area relative to the myofibril area (Figure 2F, *p*<0.05). Key components of the mitochondrial fusion system are mitofusin (Mfn) and optic atrophy 1 (OPA1) proteins. Mfn is located at the outer mitochondrial membrane, whereas OPA1 locates at the inner membrane. These two proteins interact together to promote the organelle fusion. Analysis of gene expression of both *Mfn* and *OPA1* genes in *A. aegypti* FM revealed a significant increase in their expression 24 h ABM (Figure 2G, *p*<0.05). Therefore, reduced organelle density and increased area, as showed by morphometric analysis was corroborated by the gene expression pattern of the fusion proteins and led us to conclude that blood feeding induces mitochondrial fusion.

### Blood-Feeding Induces a Time-Dependent Reversible Inhibition of Mitochondrial H_2_O_2_ Generation

**Figure 3:**
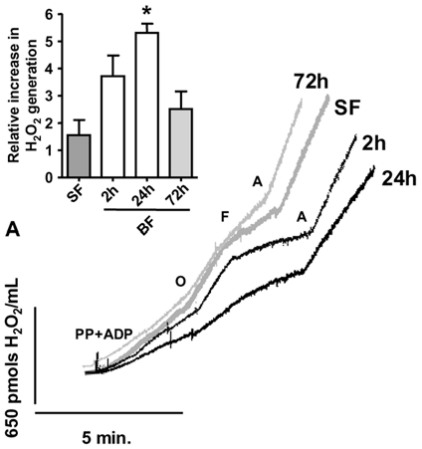
Blood feeding induces a time-dependent reversible inhibition of mitochondrial H_2_O_2_ generation. Representative traces of H_2_O_2_ formation from sugar fed (SF, thick gray line) and three different times ABM (2 h, 24 h and 72 h) of *A. aegypti* FM mitochondria using 10 mM of the substrates pyruvate-proline. Additions were 10 mM pyruvate-proline and 1 mM ADP (PP + ADP). The non-phosphorylating state 4-like respiration was induced by the addition of 4 µg/mL oligomycin (O). Uncoupled respiration was measured after the addition of up to 5 µM FCCP (F). To assess the maximum H_2_O_2_ generation rate, 4 µg/mL of antimycin a (A) was added after FCCP. ***The inset***. The maximum capacity of mitochondrial H_2_O_2_ formation was measured by the addition of antimycin a after FCCP-stimulated uncoupled respiration. Values were expressed as the relative increase (folds) of antimycin a on H_2_O_2_ formation compared to FCCP and represent mean ± SEM. Statistical analyses between groups were performed using ANOVA and *a posteriori* Tukey's test. (* *p*<0.01, relative to SF).

A potential consequence of mitochondrial fusion could be a reduction in H_2_O_2_ generation, as demonstrated by other models [6], [7]. To determine whether the mitochondrial functional (Tables 1 and 2 as well as Figure S1 and S3,) and morphological (Figure 2) changes promoted by BF could affect reactive oxygen species (ROS) generation, we measured H_2_O_2_ production in mitochondrial preparations from SF and BF mosquitoes using pyr/pro as substrates (Figure 3). Comparing all the traces shown in Figure 3, we can observe that, similar to other insects, *A. aegypti* mitochondria were capable of producing H_2_O_2_ induced by NAD^+^-linked substrates in a protonmotive force-dependent fashion. In samples from BF mosquitoes a reduction in mitochondrial H_2_O_2_ production took place as early as 2 h ABM. However, this effect was further potentiated at 24 h ABM when H_2_O_2_ generation, in both states 3 and 4-like, were significantly (*p*<0.001) reduced (Table 3). Reduction of mitochondrial H_2_O_2_ generation caused by BF was also observed when G3P was used as substrate (Figure S4). Interestingly, when blood digestion was completed (at 72 h ABM), mitochondrial H_2_O_2_ formation was restored to levels indistinguishable from those exhibited by SF. Although not statistically significant, there was also a clear transient reduction in H_2_O_2_ generation during the digestive process in the presence of the uncoupler FCCP (Table 3). The subsequent addition of the complex III inhibitor antimycin a, a procedure that maximally stimulates H_2_O_2_ production [1], results in similar levels of H_2_O_2_ in all groups (Figure 3 and table 3). Curiously, BF significantly (*p*<0.01) increased the relative effect of antimycin a on the induction of H_2_O_2_ generation, particularly at 24 h ABM, when a 5.3 fold increase was observed compared to H_2_O_2_ production rates in the uncoupled state (Figure 3, *inset*). This result shows that the potential to generate ROS is still present in the mitochondria of BF insects, indicating that a regulatory mechanism is acting to prevent ROS formation.

**Table 3:** Rates of hydrogen peroxide generation in FM mitochondria from SF and BF *A. aegypti* along the blood digestion process oxidizing pyruvate and proline.

	PP + ADP	% red.	Oligo	% red.	FCCP	% red.	AA	% red.
**SF**	419.1±89.6 (n = 9)	_	714.1±124.3 (n = 9)	_	368.1±104.4 (n = 9)	_	827.4±205.3 (n = 9)	_
**BF 2 h**	341.4±8.43 (n = 11)	18,50%	563.1±32.7 (n = 9)	21,10%	193.9±30.6 (n = 11)	47,30%	721.1±146.0 (n = 3)	12,85%
**BF 24 h**	203.0±15.5 (n = 17) *c*	51,60%	281.6±55.7 (n = 7) *c*	60,60%	128.2±20.1 (n = 7)	65,20%	681.1±42.8 (n = 4)	17,68%
**BF 72 h**	268.0±42.21 (n = 12)	36,10%	426.5±70.9 (n = 11)	40,20%	187.4±48.9 (n = 13) *b*	49,10%	634.6±145.8 (n = 10)	23,30%

H_2_O_2_ formation in SF and in BF three different times ABM (2 h, 24 h and 72 h) *A. aegypti* FM mitochondria was determined. Values were expressed as mean ± SEM of pmols H_2_O_2_ generated/min/mg protein in four different mitochondrial metabolic states using: 10 mM pyruvate + proline + 1 mM ADP (PP+ADP), followed by the addition 4 µg/mL oligomycin (Oligo) and 5 µM FCCP. To assess the maximum H_2_O_2_ generation rates, 4 µg/mL antimycin a (A) were added after FCCP. Inhibition of H_2_O_2_ formation induced by BF were expressed as percentage relative to their respective metabolic states in SF. Statistical analyses between groups were performed by using ANOVA and *a posteriori* Tukey's test. *^b^*, *p*<0.01; *c*, *p*<0.001, relative to SF.

## Discussion

**Figure 4:**
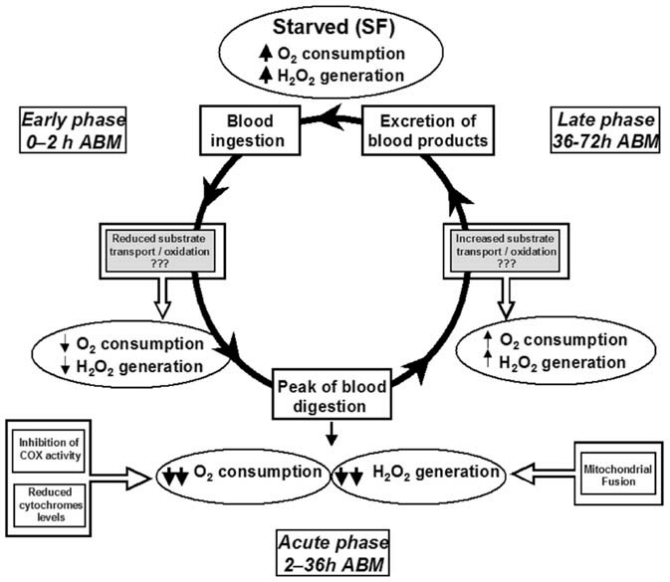
Schematic representation of the effects promoted by blood feeding on *A. aegypti* FM mitochondria. In starved insects (sugar-fed, SF) the oxygen consumption and H_2_O_2_ generation rates the levels of cytochromes *c* and *a* + *a*_3_, as well as the activity of COX, are high. Also, in SF mosquitoes mitochondria are less fused. Ingestion of vertebrate blood triggers a cascade of events that change over time after blood meal (ABM), which takes around 72 h (inner bold black circle). Our results indicate three distinct phases in which changes in mitochondrial structure and function occurs over time in *A. aegypti* FM. These events paralleled blood intake and digestion. First is the *“early”* phase, which occurs in the first two hours ABM and is characterized by a mild reduction in both oxygen consumption and H_2_O_2_ generation rates. We speculated that midgut distention induced the release of factors that would reduce substrate transport/oxidation, which mechanistically could explain these events [58]. In the *“acute”* phase, between 2 h and 36 h ABM, the effects of BF on mitochondrial structure and function are further potentiated being oxygen consumption and H_2_O_2_ generation drastically reduced. At this phase, COX activity and cytochrome levels were significantly reduced and mitochondria are more fused. Finally, at the “*late*” phase, between 36 h and 72 h ABM, most of the mitochondrial changes observed here are restored to levels similar to SF.

The pioneer studies of Gonda and Avi-Dor were the first to show oxygen consumption and phosphate esterification induced by TCA cycle substrates in a particulated fraction from whole SF *A. aegypti* [38], [39]. Since then, a number of physiological and metabolic effects triggered by blood meal were identified in hematophagous vectors [31]–[36], [40]–[44]. Despite the medical importance, a detailed evaluation of mitochondrial function in a blood-feeding organism was lacking. The present report represents the first assessment of mitochondrial structure and function in a hematophagous model during the entire blood digestion cycle. The data presented here indicates that blood feeding induces reversible functional changes in mitochondria of *A. aegypti* FM, which occur in parallel to the course of blood digestion. A summary of the observed effects induced by blood meal on FM mitochondria is schematically depicted in Figure 4.

We propose three distinct phases of mitochondrial structure and function changes which were identified during blood meal digestion in *A. aegypti*. The first one is the *“early”* phase (up to 2 h ABM), when both respiration and H_2_O_2_ generation at state 3 were slightly reduced by 27.5% and 18%, respectively (Tables 2 and 3). Possibly, signals released by the large distention of the midgut may be required at that time to promote the rapid changes observed in mitochondrial function. The *“acute”* phase (between 2 h and 36 h) represents the time period, which the effects of BF on mitochondrial structure and function were further potentiated with oxygen consumption and H_2_O_2_ generation decreased by 68.7% and 51.6% at state 3, respectively (Tables 2 and 3). Interestingly, both parameters were reversibly inhibited, reaching their lowest levels at 24 h ABM (Figure S3 and S4, Tables 2 and 3) when the digestion of host blood is at its maximum [32]. In this sense, changes in the hemolymphatic levels of the steroid hormone ecdysone and the terpenoid juvenile hormone (JH) could be involved in repressing oxygen consumption and H_2_O_2_ production observed at this time point. It is known that ecdysone levels increase whereas JH drops in the first hours of blood digestion, reaching the maximum changes at 24 h ABM [44]. It is important to note that the observed alterations promoted by blood feeding were not due to an overall mitochondrial shutdown since the activity of some inner membrane complexes, such as the F_1_F_o_ ATPase, complex I-III and G3PDH-complex III remained unchanged (Figure S1). Rather, our data shows that BF induces selective changes on FM mitochondrial function since cytochrome *c* oxidase activity and the contents of cytochromes *c* and *a*+*a*_3_ were significantly reduced 24 h ABM (Figure 1). We speculate that mitochondrial fusion (Figure 2) may also be involved in the reduction of oxygen consumption and, especially, on the H_2_O_2_ production observed at 24 h ABM. Finally, in the “*late*” phase (between 36 h and 72 h), most of the mitochondrial aspects studied here were restored to levels comparable to SF. Particularly relevant to notice is that mitochondrial oxygen consumption is reversibly decreased in such a way that addition of the uncoupler FCCP did not restore the respiratory rate to the levels exhibited by SF insects (Figure S1 and S3; Tables 1 and 2). This indicates that either substrate transport/oxidation is decreased or ETC is somehow impaired. However, it is unlikely that reductions in oxygen consumption observed in BF insects would be a consequence of nitric oxide-induced cytochrome *c* oxidase inhibition [45] since *Aedes aegypti* does not code for a canonical nitric oxide synthase (Silva-Neto, MAC – personal communication). Nevertheless, since cytochrome *c* oxidase activity is significantly reduced 24 h ABM this indicates that reduction in electrons flow at this ETC complex affects respiratory rates, despite the increase in electron turnover (Figure 1C). Moreover, at this time point, mitochondria from BF mosquitoes were in a more fused state (Figure 2) an event that was preceded by a reduction in H_2_O_2_ generation (Figure 3 and Table 3). Taken together, our data shows that mitochondrial remodeling is part of a finely regulated program triggered by blood feeding in *A. aegypti*, which may represent an important adaptation to hematophagy.

A hypothesis previously raised by our group postulated that a reduction in mitochondrial function, observed in several blood feeding parasites would represent an adaptive response to avoid the synergistic effects of mitochondrial ROS generation and pro-oxidant products of blood digestion [46]. We observed here that significant reductions of both oxygen consumption (Figure S1 and S3; Tables 1 and 2) and H_2_O_2_ production (Figure 3, Figure S4 and Table 3) occurred at 24 h ABM when blood digestion is at its maximum [32]. In this context, trypsin is the major endoprotease in *A. aegypti* [42] that breaks down blood proteins into aminoacids used both as energy and carbon skeleton sources for egg maturation and to replenish maternal reserves. Trypsin expression and secretion behaves biphasically along the blood-feeding process [42]. The “*early*” form of this enzyme appears in the midgut in the first 4–6 h ABM, whereas the “*late*” form, that is responsible for most of the proteolytic activity in midgut during blood digestion, occurs between 8 h and 36 h ABM. As changes in mitochondrial oxygen consumption in *A. aegypti* began almost immediately ABM, even 15 minutes after feeding (Figure S3, Table 2) when blood digestion has not started yet, the involvement of blood digestion products on the mitochondrial changes observed here could only be considered in later times (24 h–40 h). Therefore, at this early period, alterations in mitochondrial function can be related to other signals, such as mechanical distention of the midgut. At later time points, heme and/or iron, derived from blood digestion, could reach *A. aegypti* hemolymph and act both as stressors and as signaling compounds, as it has been reported for the kissing bug *R. prolixus* [25], [47]. In this regard, previous works have demonstrated that reduction in the mitochondrial protein frataxin, affect mitochondrial iron metabolism [48] and oxidative phophorylation [49]. Whether frataxin mediates blood meal-induced mitochondrial remodeling in *A. aegypti* remains to be elucidated. Notwithstanding, recent evidence from Zhou and co-workers [50] has shown that in *A. aegypti* females most of the blood meal-derived iron is excreted and the remaining is essentially directed to the eggs. Curiously, hemolymphatic levels of iron do not significantly change over *Aedes* digestion cycle [50]. Further research is needed to determine which factors are implicated in the mitochondrial functional and morphological changes triggered by blood feeding.

Microarray analysis of whole *Anopheles gambiae* 24 h ABM revealed that 64 transcripts related to intermediary metabolism were down regulated; more specifically 28 linked to oxidative phosphorylation and 9 to glycolytic pathway [40]. A further study has shown that in *A. gambiae* 171 transcripts related to intermediary metabolism were differently expressed during blood feeding [41]. Particular interesting is the reduced expression of pyrroline-5-carboxylate reductase, an enzyme involved in proline utilization as an energy substrate during flight in *A. aegypti* [41]. Therefore, the microarray data showing down regulation of both oxidative and fermentative metabolism were interpreted as a change in energy demand by blood-engorged female, whose energy priority would be shifted towards oogenesis while the starved mosquito has to invest energy in flight to get the blood meal. The huge blood meal intake presents an enormous payload to the flight muscle since *A. aegypti* ingests, in a single meal, about 2.5 times its own body weight in blood. Afterwards, keeping a high metabolic demand in FM would compete for substrates in tissues with an emerging demand to support the oogenesis/embryogenesis (ovaries) [44]. However, some apparent discrepancies were found between the data presented here for *Aedes* and the microarray studies conducted in *Anopheles*, which showed reduced expression of genes related to oxidative phosphorylation and TCA cycle after blood feeding [40], [41]. In this sense, despite the reduced expression of glycerol-3-phosphate dehydrogenase, six subunits of NADH-ubiquinone oxidoreductase, four subunits of ubiquinol-cytochrome *c* oxidoreductase and six subunits of F_1_F_o_ATP synthase ABM [51] in *Aedes* midgut, the functional analyses conducted here shows that these mitochondrial components were not affected in FM after a blood feeding (Figure S2). On the other hand, reductions in both cytochrome *c* oxidase activity and cytochromes *a*+*a*_3_ levels (Figure 1) in *Aedes aegypti* were in agreement with reduced expression of six subunits of the mitochondrial ETC complex in *Anopheles* [40]. Nevertheless, reductions in FM mitochondrial activity and oxygen consumption may represent a strategy developed by this insect to divert substrates utilization to other tissues with increased metabolic demands involved in activities other than flight. Thus, in addition to the consequences of the redox balance on mitochondrial remodeling observed here, the present study provides a strong bioenergetic explanation of reduced locomotion observed in blood-engorged mosquitoes.

Despite the obvious role in energy and redox metabolism as well as in apoptosis, mitochondria are very dynamic organelles, varying in size and shape in different cells under diverse stimuli. Although the first evidence of mitochondrial dynamics was reported almost 40 years ago [52] the molecular mechanisms that underlie mitochondrial fission and fusion are still being elucidated [53], [54]. Mitochondrial fusion was related to protective events since the organelle can exchange damaged DNA and even rescue membrane potential [10], [55]. It has also been shown that high glucose concentrations increased both ROS production and mitochondrial fission in rat heart muscle cells [6], [7]. Inhibition of fission machinery, and consequently maintenance of fused mitochondria, abrogates ROS generation, suggesting that mitochondria fuse to control ROS production [6], [7]. Induction of mitochondrial hyperfusion in cells exposed to specific stresses results in a highly interconnected mitochondrial network, representing a novel adaptive pro-survival response against different stresses [56]. Based on the data shown in figure 2, blood-feeding in *A. aegypti* triggers signaling responses, leading to an activation of both *Mfn* and *OPA1*, and resulting in mitochondrial fusion, an event parallel to a reduction in H_2_O_2_ production (Figure 3, Table 3). However, in *A. aegypti*, observation of reduced H_2_O_2_ generation (2 h ABM, Figure 3 and Table 3) occurs prior to the increase in expression of fusion genes (14 h ABM, data not shown), indicating that mitochondrial fusion is a later event that takes place after the reduction of ROS levels. Nevertheless, recent evidence obtained in *Drosophila* has pointed out the key role of *OPA1* on reduced ROS generation, resistance to oxidative stress and lifespan [57]. This aspect deserves additional studies to further elucidate the relationship between mitochondrial redox metabolism and dynamics in *A. aegypti*.

The rate of mitochondrial H_2_O_2_ production represents the steady-state level between molecules produced by mitochondria and those that are detoxified by the antioxidant systems. After blood feeding, the transcript abundance of important antioxidant enzymes, such as catalase and superoxide dismutase, was increased in *Aedes* midgut and whole *Anopheles* mosquitoes [40], [51]. One could argue that reduced rates of H_2_O_2_ generation observed in *Aedes* FM mitochondria upon blood feeding (Figure 3, Table 3) could be explained by the increased levels of the antioxidant defenses. However, the rate of H_2_O_2_ production in isolated FM mitochondria were decreased by 18.5% and 47.3% in state 3 and FCCP-induced respiration, respectively, 2 h ABM (Figure 3 and Table 3) a time point where there is no increase in the transcript levels of antioxidant enzymes [51]. The same conclusion is reinforced by the observation that maximal H_2_O_2_ generation rates, measured after the addition of antimycin a [1], were not altered between SF and any of the BF time points (Figure 3 and Table 3). In fact, the relative increment in H_2_O_2_ generation was even increased 24 h ABM (Figure 3 *inset*) indicating that the potential to generate ROS is still present in BF mosquitoes and that increased levels of antioxidant defenses can not explain, *per se*, the transient reduction of mitochondrial H_2_O_2_ generation. We also need to consider that the microarray study conducted in *Aedes* [51] was carried out using the insect midgut and not the FM, tissues with very different functions and therefore with distinct metabolic demands. Mechanistically, reductions in mitochondrial oxygen consumption and H_2_O_2_ generation triggered by a blood meal could be explained by reduced electron flow in the ETC produced by either diminished mitochondrial substrate uptake [58] and/or oxidation.

Taken together, the results presented here show that changes in *A. aegypti* FM mitochondrial metabolism and structure are linked to blood intake and may represent an important adaptive mechanism to hematophagy. Further analysis of this system will provide insights not only into the consequences of changes in mitochondrial function induced by blood feeding on the energy and redox metabolism but also on the signaling pathways involved in these events.

## Supporting Information

Figure S1 Blood meal reduces oxygen consumption in *A. aegypti* FM mitochondria. Representative oxygen consumption traces of sugar fed (SF, solid lines) and 24 h ABM (BF, dashed lines) *A. aegypti* FM mitochondria using 10 mM of the substrates pyruvate-proline (PP) (A) and glycerol 3-phosphate (G3P) (B). The phosphorylating state 3 respiration was induced by the addition of 1 mM ADP (ADP) and is indicated by the dotted lines. The non-phosphorylating state 4-like respiration was induced by the addition of 4 µg/mL oligomycin (o). Uncoupled respiration was measured by using up to 5 µM FCCP (F).
(2.43 MB TIF)

Figure S2 Mitochondrial yield and purity do not change regardless the *A. aegypti* diet. Comparison of enzyme activities in sugar-fed (SF, gray bars) and and 24 h ABM (BF, white bars) *A. aegypti* FM mitochondria. (A) Azide-sensitive F1Fo ATPase activity was measured in frozen-thawed mitochondrial preparations (SF, n = 7 vs. BF, n = 5; *p* = 0.7803). (B) NADH-cytochrome c oxidoreductase (complexes I - III) measured as the rotenone-sensitive rate of NADH-stimulated reduction of ferricytochrome c at 550 nm (SF, n = 17 vs. BF, n = 7; *p* = 0.4447). (C) G3P-cytochrome c oxidoreductase (complexes II - III), measured as the antimycin a-sensitive rate of G3P-stimulated reduction of ferricytochrome c at 550 nm (SF, n = 14 vs. BF, n = 7; *p* = 0.3629). Bars represent mean ± SEM. Statistical analyses were performed by using the Student's t-test.
(0.02 MB PDF)

Figure S3 Blood meal induces a time-dependent reversible inhibition of mitochondrial oxygen consumption. Representative traces of oxygen consumption in sugar fed (SF, solid black line) and five different times ABM (0.25 h, 2 h, 24 h, 40 h and 72 h - solid grey, dashed and dotted lines) *A. aegypti* FM mitochondria using 10 mM of the substrates pyruvate-proline (PP). The phosphorylating state 3 respiration was induced by the addition of 1 mM ADP (ADP), and the non-phosphorylating state 4-like respiration was induced by the addition of 4 µg/mL oligomycin (O). Uncoupled respiration was measured by using 5 µM FCCP (F).
(2.43 MB TIF)

Figure S4 Blood-feeding reduces mitochondrial H_2_O_2_ generation induced by G3P. Representative H_2_O_2_ formation traces of sugar fed (SF, gray line) and 24 h ABM (BF, black line) *A. aegypti* FM mitochondria using 10 mM of glycerol 3-phosphate + 1 mM ADP (G3P+ADP).
(2.43 MB TIF)
