# Supplementary figures and images for: Blood-Feeding Induces Reversible Functional Changes in Flight Muscle Mitochondria of Aedes aegypti Mosquito
Source: PLoS One. 2009 Nov 16;4(11):e7854. doi: 10.1371/journal.pone.0007854 (PMC2773413; doi:10.1371/journal.pone.0007854)

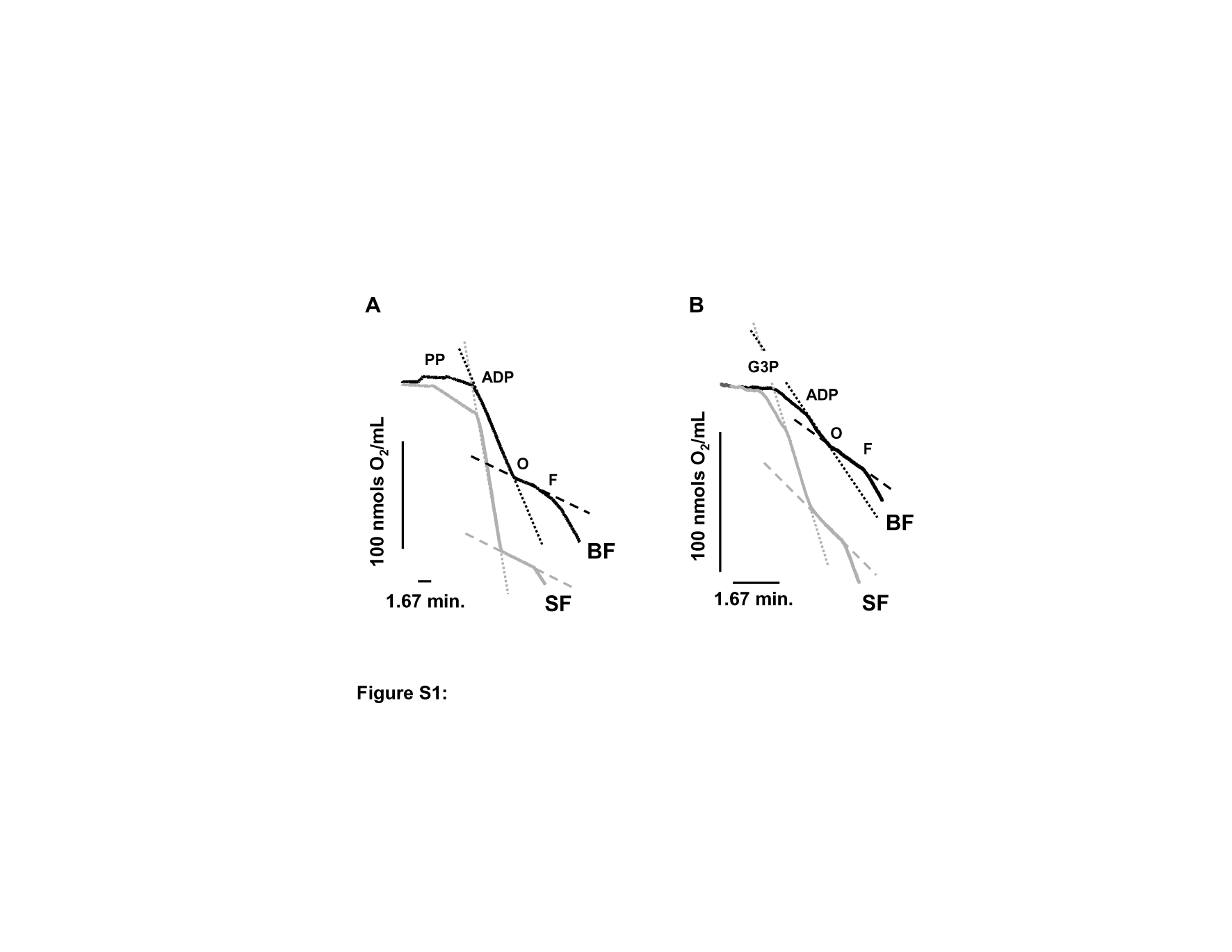

Supplement: Figure S1 — Blood meal reduces oxygen consumption in A. aegypti FM mitochondria. Representative oxygen consumption traces of sugar fed (SF, solid lines) and 24 h ABM (BF, dashed lines) A. aegypti FM mitochondria using 10 mM of the substrates pyruvate-proline (PP) (A) and glycerol 3-phosphate (G3P) (B). The phosphorylating state 3 respiration was induced by the addition of 1 mM ADP (ADP) and is indicated by the dotted lines. The non-phosphorylating state 4-like respiration was induced by the addition of 4 µg/mL oligomycin (o). Uncoupled respiration was measured by using up to 5 µM FCCP (F). (2.43 MB TIF) [file pone.0007854.s001.tif]

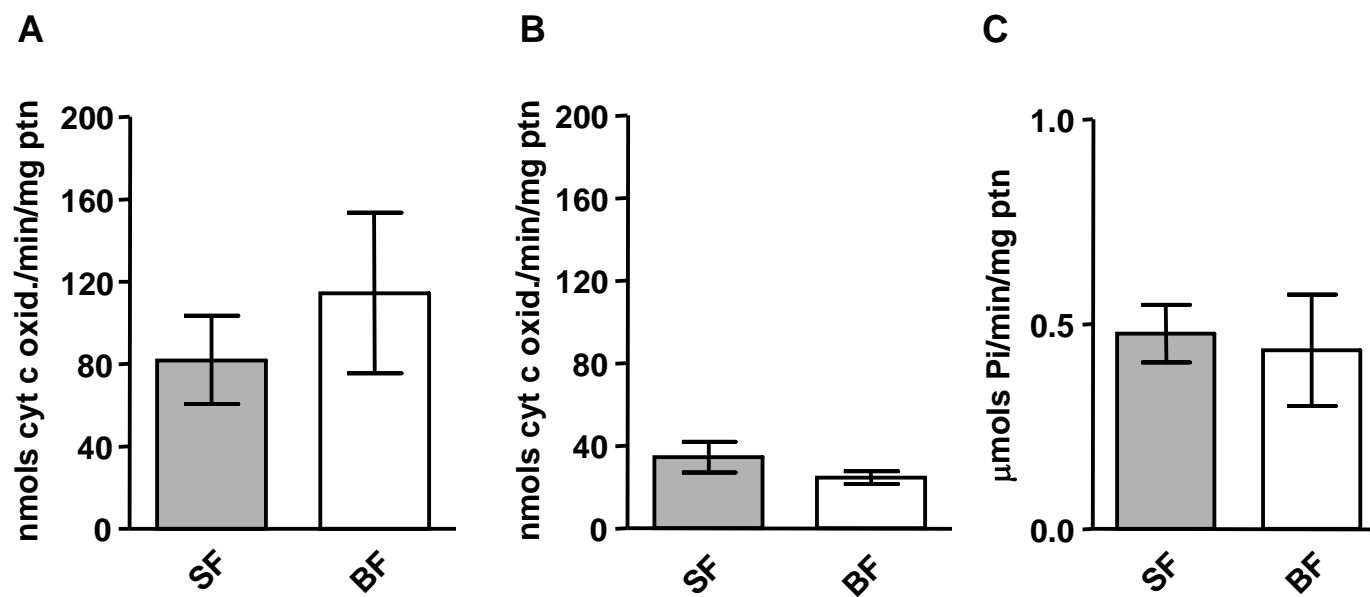

Figure S1:

Supplement: Figure S2 — Mitochondrial yield and purity do not change regardless the A. aegypti diet. Comparison of enzyme activities in sugar-fed (SF, gray bars) and and 24 h ABM (BF, white bars) A. aegypti FM mitochondria. (A) Azide-sensitive F1Fo ATPase activity was measured in frozen-thawed mitochondrial preparations (SF, n = 7 vs. BF, n = 5; p = 0.7803). (B) NADH-cytochrome c oxidoreductase (complexes I - III) measured as the rotenone-sensitive rate of NADH-stimulated reduction of ferricytochrome c at 550 nm (SF, n = 17 vs. BF, n = 7; p = 0.4447). (C) G3P-cytochrome c oxidoreductase (complexes II - III), measured as the antimycin a-sensitive rate of G3P-stimulated reduction of ferricytochrome c at 550 nm (SF, n = 14 vs. BF, n = 7; p = 0.3629). Bars represent mean ± SEM. Statistical analyses were performed by using the Student's t-test. (0.02 MB PDF) [file pone.0007854.s002.pdf]

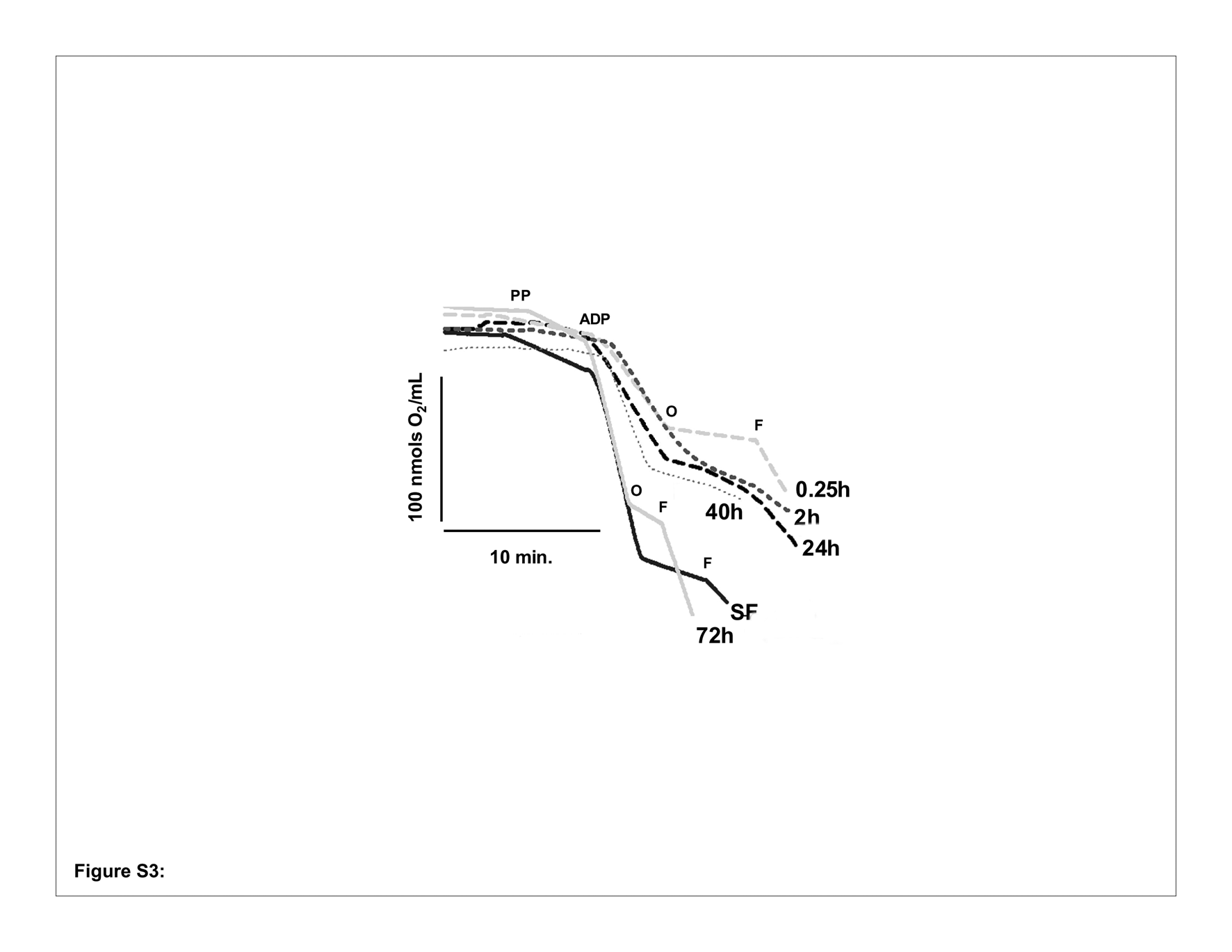

Supplement: Figure S3 — Blood meal induces a time-dependent reversible inhibition of mitochondrial oxygen consumption. Representative traces of oxygen consumption in sugar fed (SF, solid black line) and five different times ABM (0.25 h, 2 h, 24 h, 40 h and 72 h - solid grey, dashed and dotted lines) A. aegypti FM mitochondria using 10 mM of the substrates pyruvate-proline (PP). The phosphorylating state 3 respiration was induced by the addition of 1 mM ADP (ADP), and the non-phosphorylating state 4-like respiration was induced by the addition of 4 µg/mL oligomycin (O). Uncoupled respiration was measured by using 5 µM FCCP (F). (2.43 MB TIF) [file pone.0007854.s003.tif]

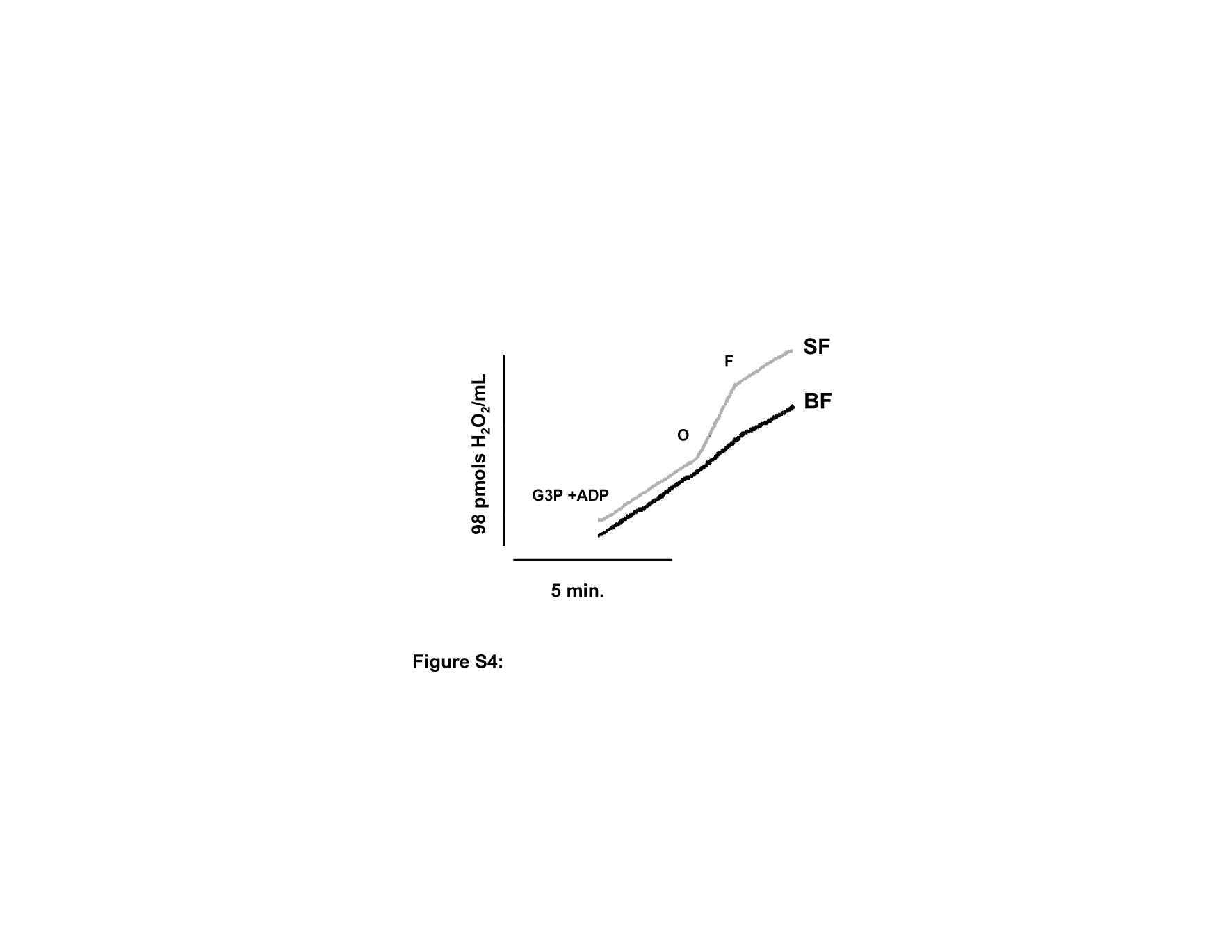

Supplement: Figure S4 — Blood-feeding reduces mitochondrial H2O2 generation induced by G3P. Representative H2O2 formation traces of sugar fed (SF, gray line) and 24 h ABM (BF, black line) A. aegypti FM mitochondria using 10 mM of glycerol 3-phosphate + 1 mM ADP (G3P+ADP). (2.43 MB TIF) [file pone.0007854.s004.tif]
